# Are we really preventing lung collapse with APRV?

**DOI:** 10.1186/s13054-019-2463-0

**Published:** 2019-05-16

**Authors:** Ryota Sato, Natsumi Hamahata, Ehab G. Daoud

**Affiliations:** 1 Department of Internal Medicine, John A. Burns School of Medicine, University of Hawaii at Manoa, 1356 Lusitana Street, 7th floor, Honolulu, HI 96813 USA; 2 Respiratory Care Program, Kapiolani Community College, Honolulu, HI USA; 3 Critical Care Department, Kuakini Medical Center, Honolulu, HI USA

**Keywords:** Airway pressure release ventilation, APRV, Trans-pulmonary pressure, Esophageal balloon

Letter to the editor

APRV is an inverse ratio, pressure-controlled, intermittent mandatory ventilation without the restriction of spontaneous breathing, and it is based on the principle of the open-lung approach [1]. A recent meta-analysis reported beneficial effects of APRV on ventilator-free days and in-hospital mortality in acute hypoxic respiratory failure; however, the quality of evidence was low [2]. Therefore, it has been still controversial whether APRV is truly beneficial or not.

The main settings of APRV include *P*_High_, *P*_Low_, *T*_High_, and *T*_Low_. There are numerous suggested ways for setting *T*_Low_ to prevent alveolar collapse, e.g., setting *T*_Low_ to create auto-PEEP empirically in a range of 0.2–0.8 s or to achieve 50–75% of peak expiratory flow or according to a certain time constant, and for a certain volume per release [1]. However, it has been unclear whether APRV truly stabilizes the alveoli and reduces lung stress and strain [3] since it is uncertain how much auto-PEEP we are creating and whether it is enough to prevent alveolar collapse especially in the setting of elevated alveolar elastance.

The measurement of trans-pulmonary pressure (*P*_TP_) using an esophageal balloon is a physiological way to detect and prevent lung stress and strain. Using that strategy has shown improved oxygenation and compliance, and trend towards improved mortality [4].

Recently, we reported that *P*_TP_ in patients undergoing APRV was in the negative values (< 0 cmH_2_O) at all levels (0.1 to 0.8 s) of *T*_Low_ suggesting the alveolar collapse but was actually positive after the application of an end-expiratory occlusion maneuver to measure total and auto-PEEP (the gold standard for measuring total PEEP) [5]. This case report demonstrates a new method to adjust *T*_Low_, also highlights the need to quantify the actual total PEEP at the end of T_Low_, and enforces the beneficial knowledge gained by esophageal balloon in APRV. The expiratory flow decay and the resultant auto-PEEP during APRV are variable among different ventilator manufacturer [1], and using a time constant may not be reliable as well. Recently, it was suggested that the use of tracheal pressure at the end of *T*_Low_ as the amount of auto-PEEP might not be accurate either [3].

In conclusion, without the knowledge of end release trans-pulmonary pressure, we are driving blind not knowing how often we are causing alveolar collapse with APRV. Therefore, there is an emergent need for more research about its settings given its rising popularity.

## Abbreviations

APRV: Airway pressure release ventilation
Auto-PEEP: Intrinsic PEEP
PEEP: Positive end-expiratory pressure
PEF: Peak expiratory flow
*P*_High_: The value of the high pressure (inspiratory pressure)
*P*_Low_: The value of the low pressure (release pressure)
*P*_TP_: Trans-pulmonary pressure
*T*_High_: Time spent at high-pressure phase
*T*_Low_: Time spent at low-pressure phase

## Authors’ information

RS and NH are internal medicine residents in the USA. ED is a board-certified intensivist in the USA.
